# A Feline Semiochemical Composition Influences the Cat’s Toileting Location Choice

**DOI:** 10.3390/ani12070938

**Published:** 2022-04-06

**Authors:** Naïma Kasbaoui, Míriam Marcet-Rius, Cécile Bienboire-Frosini, Fanny Menuge, Philippe Monneret, Estelle Descout, Alessandro Cozzi, Patrick Pageat

**Affiliations:** 1 Animal Behaviour and Welfare Department, Research Institute in Semiochemistry and Applied Ethology (IRSEA), Quartier Salignan, 84400 Apt, France; m.marcet@group-irsea.com (M.M.-R.); f.menuge@group-irsea.com (F.M.); 2 Molecular Biology and Chemical Communication Department, Research Institute in Semiochemistry and Applied Ethology (IRSEA), Quartier Salignan, 84400 Apt, France; c.frosini@group-irsea.com; 3 Animal Experimentation Service, Research Institute in Semiochemistry and Applied Ethology (IRSEA), Quartier Salignan, 84400 Apt, France; p.monneret@group-irsea.com; 4 Data Management and Statistics Service, Research Institute in Semiochemistry and Applied Ethology (IRSEA), Quartier Salignan, 84400 Apt, France; e.descout@group-irsea.com; 5 Research and Education Board, Research Institute in Semiochemistry and Applied Ethology (IRSEA), Quartier Salignan, 84400 Apt, France; p.pageat@group-irsea.com

**Keywords:** anal glands, cat welfare, domestic cat, elimination behavior, feces, semiochemistry, unwanted toileting, urine

## Abstract

**Simple Summary:**

Unwanted toileting is amongst the most undesirable behaviors in cats. The aim of this study was to test the effect of a chemical cue derived from cat anal glands on the elimination behavior of domestic cats tested individually. A total of 31 cats were tested for 23 h in an enriched test room where they had the choice between two litter trays, one sprayed with the composition tested and the other with the control. We measured the weight of elimination, the weight of urine, we recorded the type of elimination, and counted the urine spots and stool piles. We also looked at the duration of exploration of litter trays, and what trays the cats were choosing to toilet as a first and second choice. We demonstrated, across all parameters, that cats urinated and defecated significantly less in the litter trays where the chemical cue was sprayed. These results confirm and strengthen those of our previous study. Future studies could explore if a chemical cue derived from male cat anal glands might be used to manage unwanted toileting in cats.

**Abstract:**

Unwanted toileting is amongst the most undesirable behaviors in domestic cats and can lead to conflicts between cats and the communities they are living in. This study aimed to confirm the effect of a semiochemical composition, reconstituted volatile fraction derived from cat anal glands, on the elimination behavior of domestic cats. A total of 31 cats were tested individually, for 23 h, in a blinded randomized choice test, with two litter trays, one sprayed with the treatment and the other with the control. Parameters included elimination weight, urine only weight, the record of the elimination type and counting of urine spots and stools, exploration duration of each litter tray, and first and second choice of litter tray to eliminate. Across all parameters, cats urinated and defecated significantly less in the litter tray where the semiochemical composition was sprayed than in the litter tray where the control was sprayed (for example: elimination weight *p* < 0.0001; urine only weight *p* < 0.0001; exploration duration *p* < 0.0001, and first elimination choice *p* < 0.0001). These results demonstrate that a semiochemical composition-derived from cat anal glands significantly decreases elimination at the location where it is sprayed. Future research is warranted to explore the possibility to manage unwanted toileting using this semiochemical composition.

## 1. Introduction

Animals explore their physical and social environment and gather information to secure the necessary resources for their survival. This environment encompasses other animals, conspecifics, or other species. Gathering information about those animals, whether they are predators, conspecifics or preys, is an essential part of assessing the environment, to avoid threats and conflicts, find and select mating partners. Communication can be defined as the transmission of information from one animal to another [1] using different sensory modalities, such as visual, auditory, and chemical signals [2,3]. All animals emit volatile organic compounds (VOC), some of these compounds have evolved to become chemical signals emitted towards a receiver, either in the same species or other species [4]. Chemical communication allows signals to travel in the air or be deposited on a substrate, releasing the information they carry even when the animals are not present [5]. In a species that can sometimes adopt a solitary lifestyle [6] such as domestic cats, this is essential, as, in natural conditions (without human intervention) and except for cat colonies, conspecific encounters may not be very frequent [5].

Research has shown that several secretions such as urine, faeces and secretions from various glands can be vectors of chemical communication [7,8,9,10] and chemical compounds have been identified in urine [11,12], faeces [13,14], and anal glands [9]. In the domestic cat, the anal glands’ structure has been described [15,16] and their chemical composition explored [9,16,17]. Scent communication using secretions from anal glands have been described in several species such as the honey badger (*Mellivora capensis*) [18], spotted hyena (*Crocuta crocuta*) [19,20] and red foxes (*Vulpes vulpes*) [7,21,22,23,24] Anal gland compounds may have a role in individual recognition [13], territorial marking or reproductive advertisement [11]. The research field of linking compounds to a specific role is expanding, such as exploring the interaction of chemical compounds and bacteria present in the glands [17] or the interaction between compounds and their binding proteins [25]. Cat elimination behaviour is a complex process [26,27] that fulfils the role of excreting waste from the body and providing information about species, sex, reproductive status of conspecifics and age for male cats [13,14]. This behaviour is different from marking behaviour where the cat may spray urine, scratch surfaces [28] and rub on objects and individuals [29,30,31] as a form of chemical communication. However, both in elimination behaviour and marking behaviour, compounds are present that seem to influence behaviour. Urinary extracts for example have been shown to influence the choice of location to eliminate in outdoor cats [32]. Regarding anal glands’ secretions, they can be deposited on a substrate or are excreted on faeces. While some of the roles of chemical compounds isolated from cat anal glands have been explored [9], there is a lack of knowledge about the influence of very specific compounds can have on cat behaviour, especially on their choice of location to eliminate. Today, different repellents systems exist to manage cat inappropriate elimination, but elimination behaviour plays a crucial role for the cat and its communication. A new approach is to develop strategies to manage this behaviour, to promote human and cat cohabitation. A previous work performed in a cattery setting showed that a semiochemical composition, a reconstituted partial volatile fraction derived from the secretion of an entire mature male cat (13 years-old) anal glands, deterred cats from defecating at the location where it was sprayed [33]. The aim of our study was to use the same semiochemical composition on cats tested individually in a randomised controlled choice trial, to test its effect on cat elimination behaviour. Our hypothesis was that the semiochemical composition tested would have an influence on cat elimination behaviour and we predicted that the semiochemical composition would deter cats from defecating at the location where it is sprayed.

## 2. Materials and Methods

The study was approved by the IRSEA ethics committee (National French Ethics Committee C2EA125) under approval number CE_2019_07_CEIS_03.

### 2.1. Animals/Subjects

It was conducted on 31 cats (10 entire males, 10 entire females, 4 neutered males, and 7 neutered females, see Table 1), in an enriched test room where they were free to move. All cats were examined by a veterinarian before being included in the study and were in good health, free of chronic disease or lower urinary tract disorders.

### 2.2. Material

**Figure 1:**
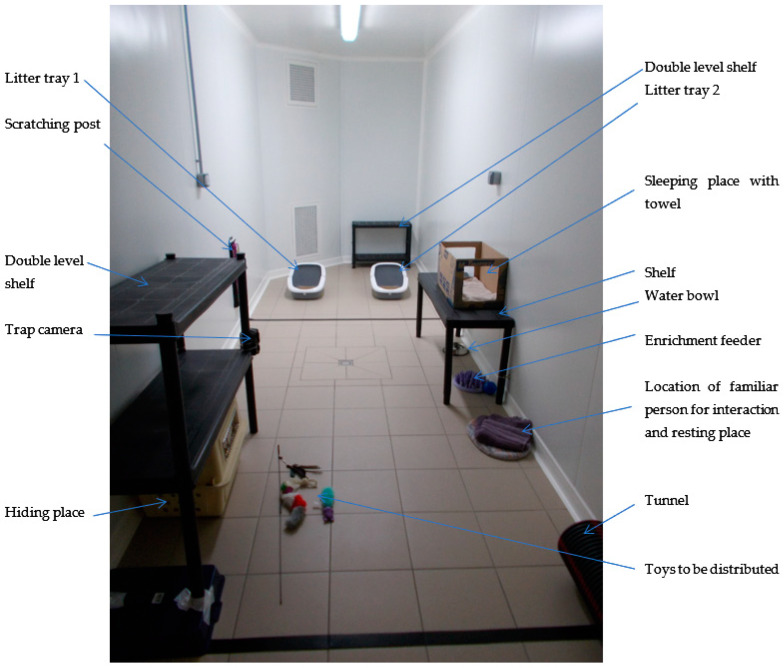
Enriched test room.

The test room (12 m^2^) contained shelves, bedding, hiding places, scratching posts, a tunnel, toys, and litter trays (Savic Aseo litter tray, L56 cm × L39 cm × H27.5 cm) (Figure 1). Dry food was provided in a puzzle feeder (Karlie flamingo NORTHMATE puzzle feeder) to match the specific needs of each cat (i.e., depending on the cat: Royal Canin Kibble VCN Adult Cat, VCN Senior Cat Stage 2 and Cat Satiety) and in a quantity sufficient for maintaining an adequate weight. Wet food (Hill’s prescription diet i/d digestive care) was used as enrichment and put in an enrichment feeder (Kong classic and Kong Extreme, size S). Water was provided ad libitum in a water bowl. The room also contained a trap camera (Coolife trap camera model H881, 16 MP 1080P HD) with motion detection sensors, focused on the area where the litter trays were positioned. 

### 2.3. Procedure

Before testing, the cats underwent a six-week progressive habituation program to ensure that they were comfortable staying alone in the test room, as they usually live in an enriched cattery environment with conspecifics. The habituation procedure consisted of first spending some time in the test room by groups of three to four cats living in the same cattery, a familiar human being present in the room. Then each cat spent some time only with a familiar human present during 20 min to provide positive interaction and opportunities to play. The familiar human then provided a small number of treats and left the room, for 20 min. The time the cat was left alone was gradually increased, from 20 min to five hours. The number of sessions depended on how each individual cat responded to being alone and was a minimum of six sessions and a maximum of ten sessions. Cats were monitored during the habituation process for signs of stress such as excessive agitation, pacing, continuous meowing, pawing/scratching at the door, refusing to eat or play and overgrooming. 32 cats entered the habituation process, and one cat was excluded from the study. All remaining cats (31) were comfortable being left alone in the test room at the end of the habituation process and didn’t show any of the aforementioned signs of stress any more. Their behaviour can be described as follows: they explored the room, played with toys, ate treats, or rested on the resting places. 

The cats were tested individually and stayed 23 h in the enriched test room, alone, except during short sessions where they positively interacted (positive contact and play) with a familiar human. Four interaction’s sessions (the first one of 30 min, then the following ones of 20 min each) were performed during the test.

The chosen experimental design was a Randomized Controlled Trial, where the cats had the choice between two litter trays (named 1 and 2) present in the test room. In each litter tray, one treatment was sprayed on the litter substrate. The treatment used was a reconstituted partial volatile fraction (named CEMS for Cat Elimination Modulation Semiochemical) of the secretion of a male cat’s anal gland, as described in Kasbaoui et al., 2022 [33]. Briefly, the treatment is composed of several volatile compounds present in the anal glands secretions obtained from an entire male cat, following their identification by GC-MS analyses. The actual treatment product is made from these same several pure chemical compounds, industrially purchased (SIGMA, Saint-Quentin-Fallavier, France), at the final concentration of 2% in a mix of ethanol and water (60:40). 

The other was the control (i.e., a mixture of ethanol and water, (60:40)).

The procedure was blinded and randomized, the treatment being applied randomly to litter tray 1 or litter tray 2 for the whole duration of the test of each individual cat. Litter trays were filled with 1.5 kg of non-agglomerant litter substrate (brand “Prop’chat NF) and treatments were sprayed (five sprays) on the litter substrate and mixed with it, according to the randomization list. Litter trays were weighed each day at the start of the experiment (Expondo digital weighing scale, model SBS-PT-40/1, with a precision of 1 g). The litter trays were then installed in the enriched test room and secured at their predetermined location with double-sided tape. The test started at 10 a.m. in the morning. The cat was brought to the test room approximately five minutes after the litter trays were put in place, to allow the ethanol to evaporate. The familiar human stayed with the cat for the interaction session during 30 min, then left the cat alone in the room, having given the Kong toy filled with wet food behind. The familiar human came back at 12:30 p.m., 2 p.m. and 4:30 p.m. for interaction sessions. After the last interaction session, the litter trays were removed from the test room in order to record data, perform the cleaning procedure, renew the litter substrates and re-apply treatments. The procedure was as follows: the litter trays were removed from the test room and weighed. The type of elimination present was recorded (urine only, stools only, or urine plus stools) and the counting of urine spots and stool piles was performed and recorded. Stools were then removed if present and the litter trays were weighed again (giving the parameter of the “urine only” weight). After all of the data were recorded, the litter substrate was discarded in a special bin. For cleaning, each litter tray was sprayed on the inside and the outside with a detergent disinfectant product that destroys liposoluble compounds (DNA02 LeVrai Professionnel, https://www.bernard.fr/, accessed 29 October 2021) and thoroughly wiped until completely clean. Then, it was sprayed a second time with water to rinse the detergent and avoid any contact between the detergent and the cats. After the litter tray was dried, 1.5 kg of fresh litter substrate was weighed and placed in the litter tray. Finally, according to the randomization list, the designated treatment was sprayed on the litter substrate and mixed with it. The litter trays were weighed to record the weight of the unused litter tray with the treatment applied and were put back in place. The whole procedure lasted less than 10 min. Then the cat was left alone for the night, and the test ended at 9 a.m. the next morning, when the cat was brought back to its cattery. Data were recorded again, and the test room was cleaned. The cleaning procedure was to remove all that was washable, then steam clean the floor and walls, spray a detergent that breaks down proteins and liposoluble compounds (VIGOR surpuissant, https://www.bernard.fr/, accessed 29 October 2021), then rinse the detergent with water, mop the floor, and wait 10 min for the room to dry completely before preparing the room for the next test. 

The parameters studied included total elimination weight (urine plus stools, which was calculated by subtracting the weight of the unused litter tray with the treatment from the weight of the used litter tray), urine weight (which was calculated by subtracting the weight of the unused litter tray from the weight of the used litter tray without stools), type of elimination (0 = no elimination, 1 = urine only, 2 = stools only, 3 = urine plus stools), and frequency of urine spots and stools (counting each urine spot and stool pile for each cat). The videos recorded were exported twice a day and stored. Video analysis allowed to study the exploration duration of each litter tray (i.e., time when the cat sniffed the litter tray, had his head inside the tray, or scratched the litter tray) and the first and second choice of litter tray to eliminate. 

### 2.4. Statistical Analysis

Data analysis was performed using SAS 9.4 software Copyright (c) 2002–2012 by SAS Institute Inc., Cary, NC, USA. The significance threshold was fixed at 5%. 

Continuous parameters (total elimination weight, urine weight, and exploration duration) were analysed according to the treatment applied (Y and Z), the sex of cats (entire male, entire female, neutered male, and neutered female), and the treatment sex interaction. The normality of residues from raw data was verified using the UNIVARIATE procedure. If normality was verified, the different effects listed on these variables were evaluated with a GENERAL LINEAR MIXED MODEL using the MIXED procedure with “block” in the random statement. If normality was not verified, Box-Cox data transformation was performed using the TRANSREG procedure to try to obtain normality. After a Box-Cox transformation, normality was obtained and transformed data were modelled using the MIXED procedure. If there were significant differences, multiple comparisons were analysed with the TUKEY test using the LSMEANS statement.

Binary variables (first and second choices of litter tray for elimination) were analysed according to the treatment applied, the sex of cats, and the treatment × sex interaction. These effects were evaluated with a GENERALIZED LINEAR MIXED MODEL (Block being considered as a random effect) using the GLIMMIX procedure, specifying the BINARY distribution in the MODEL statement. If there were significant differences, multiple comparisons were analysed with the TUKEY test using the LSMEANS statement.

Polytomous variables (type of elimination, frequency of urine spots and stools) were analysed according to the treatment applied, the sex of cats, and the treatment × sex interaction. These effects were evaluated with the help of the GENERALIZED LINEAR MIXED MODEL (Block being considered as a random effect) through the GLIMMIX procedure, specifying the MULTINOMIAL distribution. 

## 3. Results

### 3.1. Elimination Weight

For the total elimination weight (Table 2), we found a significant effect of:

(i) **treatment** (**GLMM**; **DF =** **1**; **F =** **43.44**; ***p* < 0.0001**) where the elimination weight was significantly lower in the litter tray sprayed with CEMS than in the litter tray with the control;
(ii) **sex** (**GLMM**; **DF =** **3**; **F =** **7.48**; ***p* = 0.0009**) where the weight of elimination in females and the weight of elimination in males were significantly lower than in neutered males; and
(iii) **interaction between treatment and sex** (**GLMM**; **DF =** **3**; **F =** **4.60**; ***p* = 0.0100**).

There was a significant difference between females and neutered males (**Tukey’s test**; **DF** **= 27**; ***t* value** **= −4.28**; ***p* = 0.0011**) and males and neutered males (**Tukey’s test**; **DF** **= 27**; ***t* value** **= −4.22**, ***p* = 0.0013**). Regarding the significant interaction effect, there was a significant difference between CEMS and control within the neutered males (**Tukey’s test**; **DF** **= 27**; ***t* value = 5.37**; ***p* = 0.0003**) and between neutered males and males within control (**Tukey’s test**; **DF = 27**; ***t* value = −5.58**; ***p*-value = 0.0002)**.

For the weight of urine only (Table 2), we observed a significant effect of:

(i) **treatment** (**GLMM**; **DF = 1**; **F = 41.23**; ***p*-value < 0.0001**) where the weight of urine only was significantly lower in the litter tray sprayed with CEMS than in the litter tray with the control;
(ii) **sex** (**GLMM**; **DF = 3**; **F = 6.81**; ***p*-value = 0.0006**) where the weight of urine only in females and the weight of urine only in males was significantly lower than in neutered males;
(iii) **interaction between treatment and sex** (**GLMM**; **DF = 3**; **F = 4.46**; ***p*-value = 0.0072**). There was a significant difference between females and neutered males (**Tukey’s test**; **DF = 54**; ***t* value = −3.78**; ***p* = 0.0022**) and male and neutered male (**Tukey’s test**; **DF = 54**; ***t* value = −4.06**; ***p* = 0.0009**).

### 3.2. Type and Number of Eliminations

We observed a significant effect of treatment for the type of elimination (**GLMM**; **DF = 1**; **F = 15.61**; ***p*-value = 0.0005**), the number of urine spots and the number of stool piles per litter tray (urine spots: **GLMM**; **DF = 1**; **F = 14.42**; ***p*-value = 0.0008**; stool piles: **GLMM**; **DF = 1**; **F = 6.66**; ***p*-value = 0.0152**). Cats significantly chose to eliminate less (score of elimination) in the litter tray sprayed with CEMS than in the litter tray sprayed with the control. They also urinated and defecated significantly less in the litter tray sprayed with CEMS than in the litter tray sprayed with the control (Table 3).

### 3.3. Exploration of the Litter Trays and Choice of Litter Tray to Eliminate

Cats explored the litter tray with CEMS significantly less than the litter tray with the control (mean ± SE: CEMS 58.16 ± 8.04 s versus control 132.56 ± 13.60 s; **GLMM**; **DF = 1**, **F = 27.86**; ***p*-value < 0.0001**) and chose the litter tray with the control to eliminate significantly more than the litter sprayed with CEMS, for the first choice (**GLMM**; **DF = 1**; **F = 100.33**; ***p*-value < 0.0001**) and second choice (**GLMM**; **DF = 1**; **F = 5.24**; ***p*-value = 0.0293**) of elimination.

For the first choice, a significant difference in the sex effect is observed (**GLMM**; **DF = 3**; **F = 23.49**; ***p*-value = <0.0001**). No significant difference in the sex effect is found for the second choice (**GLMM**; **DF = 3**, **F = 0.02**; ***p*-value = 0.9949**).

These results showed that cats avoided the litter tray sprayed with the CEMS treatment (Table 4). 

During the study, two cats out of 31 urinated outside the litter tray (6% of the population tested).

Taking into account all results, cats significantly chose to eliminate (urinate or defecate) in the litter tray sprayed with the control rather than the litter tray sprayed with CEMS. 

## 4. Discussion

The aim of this study was to assess the effect of a semiochemical composition, reconstituted volatile fraction derived from male cat anal glands (of which some effect had been shown in a previous study [33]), on the elimination behaviour of cats tested individually, in a randomised controlled choice trial. We showed that cats urinated and defecated significantly less in a litter tray sprayed with the treatment CEMS than in a litter tray sprayed with the control. They also explored the litter tray treated with CEMS significantly less than the one with the control, and their first and second choice for elimination was significantly the litter tray sprayed with the control treatment. There was a significant effect of sex (females versus neutered males and males versus neutered males) for the elimination weights (urine plus stools and urine only) where neutered male produced a higher weight of total elimination and a higher weight of urine only then both females and males.

### 4.1. Effect of Treatment

Our study showed that cats urinated and defecated significantly less in the litter trays sprayed with the CEMS treatment, across all of the elimination behaviour parameters: elimination weight, type of elimination, urines spots, and stools piles. These results confirm the ones of our previous study regarding defecation and give more data about urination (urine weight and urine frequency). 

The CEMS treatment also had a significant effect on the first and second choice of litter tray for elimination, with the control litter trays being significantly chosen for the first elimination event and for the second elimination event. Some cats even always chose the control litter tray. So, the CEMS treatment seems to have an aversive effect and significantly deter cats from choosing the litter tray where it is sprayed. This is confirmed by the parameter “exploration duration of the litter trays”, with the litter tray sprayed with the CEMS treatment being explored significantly less than the litter tray with the control treatment. The exploration duration parameters and the choice of elimination parameters were co-dependent, as the exploration of the litter tray included the scratching of the litter substrate. 

Urine, feces, and sebaceous glandular secretions contains chemical compounds that are vectors of chemical communication [7,8]. Anal gland secretions can be excreted in cat urine [7] and also on cat feces [9]. When allowed outside and living in a colony, cats bury their feces in their core home range and tend to bury them less in the peripheral areas of their home range [8]. Entire male cats also sometimes do not cover their feces as well [8] and cat feces contain the chemical basis of species, sex, and individual recognition [13]. The semiochemical composition tested in this study is specific to an entire male cat. Therefore, it is possible that the avoidance behaviour was triggered by this semiochemical composition, considered as a territorial scent marking. 

### 4.2. Effect of Sex

There was a significant effect of sex in three out of eight parameters. The first effects were on the elimination weight (i.e., total elimination weight (urine plus stools) and urine only weight). For both parameters, the statistical analysis showed that there was a difference between female and neutered males, and males and neutered males. From a previous study of cats in our cattery [33], we showed that neutered male cats were also significantly heavier than female cats. Neutered male cats are also older than male and female cats. As water intake and urine production can be influenced by cat weight [34], it is possible that the elimination weight of neutered male cats, both depending on their average weight and their condition, could be higher than that of male and female cats that were much younger (approximately three years old) and for the females, significantly lighter. This hypothesis is also supported by the fact that there was no effect of sex on the number of urine spots. Therefore, neutered males did not urinate more frequently, but produced more urine during their urination. 

The avoidance effect depended on the cat, and in our study, it was present in the four sex categories tested (entire males and females, neutered males, and females), despite significant differences between them. During the study, no cats showed signs of stress making necessary to stop the test. A few cats were more sensitive than others and refused to use either litter tray and sprayed and urinated outside the litter tray. It is possible that the perception of this semiochemical composition was perceived as stressful for the most sensitive cats [35] in our enclosed study setting. However, in the test conditions, the cats could be away from the litter trays, but could not leave the room. In real life conditions, cats could freely leave the location where the CEMS treatment would be applied, rendering less likely the possibility to be stressed by continuous exposure to a signal triggering avoidance. Moreover, chemical signals are a part of the cats’ daily lives [5] so the signal would be, in real life, one signal present in a wealth of other chemical signals, and part of the cats’ natural environment. 

## 5. Conclusions

We demonstrated that a semiochemical composition, reconstituted volatile fraction derived from cat anal glands, deterred the subjects from defecating and urinating in the litter tray where it was sprayed. This semiochemical composition does not prevent the elimination of the cat but could promote redirection of elimination. Future research is warranted to explore the possibility of using this semiochemical composition to help manage unwanted toileting in real life conditions and if this composition, while triggering avoidance, may also modulate other behaviours of cats and/or have stressful effects on cats. 

## Figures and Tables

**Table 1:** Cats participating in the experiment, cat sexes, and ages. Entire male and female cats were born within the same two weeks so the age presented is the average age for entire male and entire female cats.

Cat Name	Cat Sexe	Cat Age
Altesse	Entire female N = 10	2 years 10 months
Biscotte		
Brioche		
Choco		
Cannelle		
Gaia		
Mia		
Xarra		
Xena		
Venus		
Matisse	Entire male N = 10	2 years 10 months
Merlin		
Misty		
Next		
Simba		
Spicy		
Willy		
Woody		
Ying		
Yang		
Batcat	Neutered male N = 4	13 years 10 months
Encre		13 years 9 months
Garfield		8 years 8 months
Guinness		8 years 8 months
Edelweiss	Neutered female N = 7	11 years 9 months
Hermine		8 years
Perle		15 years
Rose		14 years 11 months
Corona		8 years 8 months
Elvira		11 years 9 months
Guimauve		8 years 10 months

**Table 2:** Means and standard error of the total elimination weight in kilograms and the urine only weight in kilograms, according to treatment and sex.

Variable	Effect		Mean	SE
Total elimination weight	Treatment	CEMS	0.02	0.00
		Control	0.07	0.01
	Sex	Entire male	0.04	0.01
		Entire female	0.03	0.01
		Neutered male	0.09	0.03
		Neutered female	0.06	0.01
Urine only weight	Treatment	CEMS	0.02	0.00
		Control	0.05	0.01
	Sex	Entire male	0.03	0.00
		Entire female	0.03	0.01
		Neutered male	0.07	0.02
		Neutered female	0.04	0.01

**Table 3:** Score of type of elimination, frequencies of urine spots and stool piles according to treatment.

**Variable**	**Effect**		**Score ^1^**																																																											
			**0**															**1**															**2**															**3**														
Type of elimination ^1^	Treatment	CEMS Control	17															9															1															4														
			3															12															3															13														
**Variable**	**Effect**		**Frequency ^2^**																																																											
			**0**												**1**												**2**												**3**												**4**											
Urine spots ^2^	Treatment	CEMS Control	18												9												4												0												0											
			6												9												11												4												1											
**Variable**	**Effect**		**Frequency ^2^**																																																											
			**0**																				**1**																				**2**																			
Stool piles ^2^	Treatment	CEMS Control	26																				3																				2																			
			15																				15																				1																			

^1^ For the type of elimination score 0 = no elimination; 1 = urine only; 2 = stools only; 3 = urine plus stools. ^2^ For the urine spots and stool piles, frequency represents the total daily number of urine spots and stool piles in the litter tray.

**Table 4:** Scores of first litter tray chosen to eliminate and on the second litter tray chosen to eliminate.

**Variable**	**Effect**		**Score**	
			**0**	**1**
First choice of elimination	Treatment	CEMS Control	26	5
			7	24
**Variable**	**Effect**		**Score**	
			**0**	**1**
Second choice of elimination	Treatment	CEMS Control	23	8
			14	17

Score 0 = litter tray not chosen; 1 = litter tray chose; Given the binomial nature of these data (choices represented by 1 and 0), differences in numbers could be partially explained by differences in choices, not only by differences in numbers of cats.

## Data Availability

The data presented in this study are available on request from a.cozzi@group-irsea.com. The data are not publicly available due to potential patent pending.

## References

[1] Green S., Marler P., Marler P., Vandenbergh J.G. (1979). The analysis of animal communication. Social Behavior and Communication.

[2] Higham J.P., Hebets E.A. (2013). An introduction to multimodal communication. Behav. Ecol. Sociobiol..

[3] Hebets E.A., Barron A.B., Balakrishnan C.N., Hauber M.E., Mason P.H., Hoke K.L. (2016). A systems approach to animal communication. Proc. R. Soc. B Biol. Sci..

[4] Wyatt T.D. (2014). Pheromones and Animal Behavior: Chemical Signals and Signatures.

[5] Shreve K.R.V., Udell M.A. (2017). Stress, security, and scent: The influence of chemical signals on the social lives of domestic cats and implications for applied settings. Appl. Anim. Behav. Sci..

[6] Page R.J.C., Ross J., Bennet D.H. (1992). A study of the home ranges, movements and behaviour of the feral cat population at Avonmouth Docks. Wildl. Res..

[7] MacDonald D.W. (1980). Patterns of scent marking with urine and faeces amongst carnivore communities. Symp. Zool. Soc. Lond..

[8] Feldman H.N. (1994). Methods of scent marking in the domestic cat. Can. J. Zool..

[9] Miyazaki T., Nishimura T., Yamashita T., Miyazaki M. (2018). Olfactory discrimination of anal sac secretions in the domestic cat and the chemical profiles of the volatile compounds. J. Ethol..

[10] Pageat P., Gaultier E. (2003). Current research in canine and feline pheromones. Vet. Clin. Small Anim..

[11] Miyazaki M., Kamiie K., Soeta S., Taira H., Yamashita T. (2003). Molecular cloning and characterization of a novel carboxylesterase-like protein that is physiologically present at high concentrations in the urine of domestic cats (*Felis catus*). Biochem. J..

[12] Westall R.G. (1953). The amino acids and other ampholytes of urine. 2. The isolation of a new sulphur-containing amino acid from cat urine. Biochem. J..

[13] Miyazaki M., Miyazaki T., Nishimura T., Hojo W., Yamashita T. (2018). The chemical basis of species, sex, and individual recognition using feces in the domestic cat. J. Chem. Ecol..

[14] Uetake K., Abumi T., Suzuki T., Hisamatsu S., Fukuda M. (2018). Volatile faecal components related to sex and age in domestic cats (*Felis catus*). J. Appl. Anim. Res..

[15] Sokolov V.E., Shabadash S.A. (1979). Histochemical characteristics of the anal sacs of the cat. Biol. Bull. Acad. Sci. USSR.

[16] Frankel J.L., Scott D.W., Erb H.N. (2008). Gross and cytological characteristics of normal feline anal-sac secretions. J. Feline Med. Surg..

[17] Yamaguchi M.S., Ganz H.H., Cho A.W., Zaw T.H., Jospin J., McCartney M.M., Davis C.E., Eisen J.A., Coil D.A. (2019). Bacteria isolated from Bengal cat (*Felis catus* × *Prionailurus bengalensis*) anal sac secretions produce volatile compounds potentially associated with animal signaling. PLoS ONE.

[18] Begg C.M., Begg K.S., Du Toit J.T., Mills M.G.L. (2003). Scent-marking behaviour of the honey badger, *Mellivora capensis* (Mustelidae), in the southern Kalahari. Anim. Behav..

[19] Drea C.M., Vignieri S.N., Kim H.S., Weldele M.L., Glickman S.E. (2002). Responses to olfactory stimuli in spotted hyenas (*Crocuta crocuts*): II. Discrimination of conspecific scent. J. Comp. Psychol..

[20] Burgener N., Dehnhard M., Hofer H., East M.L. (2009). Does anal gland scent signal identity in the spotted hyaena?. Anim. Behav..

[21] Blizard R.A., Perry G.C. (1979). Response of captive male red foxes (*Vulpes vulpes* L.) to some conspecific odors. J. Chem. Ecol..

[22] White P.J., Kreeger T.J., Tester J.R., Seal U.S. (1989). Anal-sac secretions deposited with feces by captive red foxes (*Vulpes vulpes*). J. Mammal..

[23] Arnold J., Soulsbury C.D., Harris S. (2011). Spatial and behavioral changes by red foxes (*Vulpes vulpes*) in response to artificial territory intrusion. Can. J. Zool..

[24] Albone E.S., Perry G.C. (1976). Anal sac secretion of the red fox, *Vulpes vulpes*; volatile fatty acids and diamines: Implications for a fermentation hypothesis of chemical recognition. J. Chem. Ecol..

[25] Bienboire-Frosini C., Durairaj R., Pelosi P., Pageat P. (2020). The major cat allergen Fel d 1 binds steroid and fatty acid semiochemicals: A combined in silico and in vitro study. Int. J. Mol. Sci..

[26] McGowan R.T., Ellis J.J., Bensky M.K., Martin F. (2017). The ins and outs of the litter box: A detailed ethogram of cat elimination behavior in two contrasting environments. Appl. Anim. Behav. Sci..

[27] Ellis J.J., McGowan R.T.S., Martin F. (2017). Does previous use affect litter box appeal in multi-cat households?. Behav. Process..

[28] Mengoli M., Mariti C., Cozzi A., Cestarollo E., Lafont-Lecuelle C., Pageat P., Gazzano A. (2013). Scratching behaviour and its features: A questionnaire-based study in an Italian sample of domestic cats. J. Feline Med. Surg..

[29] Mertens C. (1991). Human-cat interactions in the home setting. Anthrozoös.

[30] Cafazzo S., Natoli E. (2009). The social function of tail up in the domestic cat (*Felis silvestris catus*). Behav. Processes.

[31] Brown S.L., Bradshaw J.W., Turner D.C., Bateson P. (2014). Communication in the domestic cat: Within-and between-species. The Domestic Cat: The Biology of Its Behaviour.

[32] Miyazaki M., Nishimura T., Hojo W., Miyazaki T., Laine R.A., Yamashita T. (2017). Potential use of domestic cat (*Felis catus*) urinary extracts for manipulating the behavior of free-roaming cats and wild small felids. Appl. Anim. Behav. Sci..

[33] Kasbaoui N., Bienboire-Frosini C., Monneret P., Leclercq J., Descout E., Cozzi A., Pageat P. (2022). Influencing Elimination Location in the Domestic Cat: A Semiochemical Approach. Animals.

[34] Fritz J., Handl S. (2018). Besoins hydriques et habitudes d’abreuvement des chats. Vet. Focus.

[35] Mills D.S., Redgate S.E., Landsberg G.M. (2011). A meta-analysis of studies of treatments for feline urine spraying. PLoS ONE.

